# Redox Mechanisms in Li and Mg Batteries Containing Poly(phenanthrene quinone)/Graphene Cathodes using Operando ATR‐IR Spectroscopy

**DOI:** 10.1002/cssc.202000054

**Published:** 2020-03-19

**Authors:** Alen Vizintin, Jan Bitenc, Anja Kopač Lautar, Jože Grdadolnik, Anna Randon Vitanova, Klemen Pirnat

**Affiliations:** ^1^ National Institute of Chemistry Hajdrihova 19 1000 Ljubljana Slovenia; ^2^ Honda R&D Europe GmbH Carl-Legien Strasse 30 63703 Offenbach Germany

**Keywords:** attenuated total reflection spectroscopy, density functional theory, organic battery, poly(phenanthrene quinone), redox chemistry

## Abstract

The redox reaction mechanism of a poly(phenanthrene quinone)/graphene composite (PFQ/rGO) was investigated using operando attenuated total reflection infrared (ATR‐IR) spectroscopy during cycling of Li and Mg batteries. The reference phenanthrene quinone and the Li and Mg salts of the hydroquinone monomers were synthesized and their IR spectra were measured. Additionally, IR spectra were calculated using DFT. A comparison of all three spectra allowed us to accurately assign the C=O and C−O^−^ vibration bands and confirm the redox mechanism of the quinone/Li salt of hydroquinone, with radical anion formation as the intermediate product. PFQ/rGO also showed exceptional performance in an Mg battery: A potential of 1.8 V versus Mg/Mg^2+^, maximum capacity of 186 mAh g^−1^ (335 Wh kg^−1^ of cathode material), and high capacity retention with only 8 % drop/100 cycles. Operando ATR‐IR spectroscopy was performed in a Mg/organic system, revealing an analogous redox mechanism to a Li/organic cell.

## Graphical abstract

**Charging mechanism**: A new poly(phenanthrene quinone) cathode material shows exceptional electrochemical performance in Li and Mg batteries. The redox reactions during charge/discharge were investigated using operando attenuated total reflectance IR spectroscopy. In both battery systems, the hydroquinone salt was reversibly oxidized back to the quinone functional group.

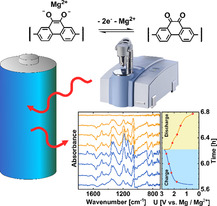

## Introduction

From wireless remotes, mobile phones to wearable electronics, battery‐powered personal devices have transformed the way we live. The development of low‐cost, safer, more powerful, and more energy‐dense battery technology will unlock innovation within the transport industry and facilitate a transition to renewables by providing flexibility in the production and consumption of energy. Most state‐of‐the‐art Li‐ion batteries employ inorganic cathodes. The cathode materials vary depending on the requirements of the application, and include lithium cobalt oxide, lithium nickel manganese cobalt oxide, lithium manganese oxide, lithium iron phosphate, and nickel cobalt aluminum oxide.^1^, ^2^ Most of these materials contain toxic or relatively rare elements (Ni, Co) and a lot of energy and high temperatures above 700 °C are required during ceramic synthesis.^3^ Their theoretical capacities are limited to approximately 200 mAh g^−1^ owing to the relatively high atomic weight of transition metals and the low number of exchanged electrons. Currently, the best material in terms of energy density is NMC 811, which has a capacity and voltage of 200 mAh g^−1^ and 3.8 V versus (vs.) Li/Li^+^, respectively, which results in a gravimetric energy density of approximately 760 Wh kg^−1^ of cathode material.^4^

Mg batteries are becoming an increasingly attractive alternative to Li‐based batteries. There are several reasons for that, including lower cost, sustainability, an improved safety profile, and higher volumetric energy density. Moreover, lithium deposits are located at some geopolitically sensitive locations and its extraction can have a big impact on the environment. On the other hand, Mg is 1000 times more abundant^5^ and can be economically produced even from seawater. As Mg does not form dendrites,^6^ it can be safely used as a metal anode with a very high volumetric capacity 3832 Ah L^−1^ (Li has a capacity of 2062 Ah L^−1^). However, there are also disadvantages such as passivation of the Mg anode, lack of suitable electrolytes,^7^ difficult insertion of the Mg^2+^ ion into inorganic materials, and slow solid‐state diffusion, which results in the lack of suitable cathodes and severely impedes the development of practical Mg batteries.^8^

On the other hand, organic materials offer a better alternative to inorganic materials in terms of versatility and compatibility with different metal counter ions,^9^, ^10^ price, gravimetric energy density, and sustainability.^11^, ^12^, ^13^, ^14^, ^15^ They can be produced from petrochemicals, biomaterials, organic waste,^3^, ^13^, ^16^, ^17^, ^18^ or even from CO_2_ as a source of carbon^19^ under low‐temperature synthesis conditions below 100 °C. Because of their low molecular mass and high number of exchanged electrons, they can reach high practical capacities of up to 600 mAh g^−1^ (Li‐rhodizonate), which could theoretically be increased up to 960 mAh g^−1^ (value for the C=O carbonyl group). This would result in a theoretical energy density of approximately 2000 Wh kg^−1^ of cathode for 2.0 V vs. Li/Li^+^ organic materials. The most common drawback of organic materials is their good solubility in organic electrolytes, which results in a fast capacity drop during cycling and self‐discharge.^12^, ^17^, ^20^ The most promising organic materials are actually redox‐active polymers with very low solubility in electrolytes. Some promising examples are poly(imides),^21^ poly(anthraquinonyl sulfide) (PAQS),^22^ poly(2,5‐dihydroxy‐1,4‐benzoquinonyl sulfide),^23^, ^24^ poly(vinylanthraquinone),^25^ polymer‐bound pyrene‐4,5,9,10‐tetraone,^26^ poly(anthraquinone) (PAQ),^27^ poly(benzenequinonyl sulfide),^28^, ^29^ and poly(diphenanthrene‐quinone substituted norbornene).^30^ Cathode or positive electrode materials based on anthraquinone, such as poly(anthraquinonyl sulfide) (PAQS)^22^ and poly(anthraquinone) (PAQ)^27^ have very stable electrochemistry, high Coulombic efficiency, good power performance (rate capability), and have been successfully exploited in both Li‐ and post Li‐battery systems.^31^, ^32^, ^33^ On the other hand, their discharge potential is only approximately 2.2 V vs. Li/Li^+^ for discharge and correspondingly lower in a Mg cell. Recently, we developed an isomer of PAQ, poly(phenanthrenequinone) (PFQ), with a potential of 2.6 V vs. Li/Li^+^.^34^ Unexpectedly, this PFQ material was insoluble in organic solvents, which resulted in high cycling stability with only 9 % drop in capacity after 500 cycles (50 mA g^−1^ or approximately 0.2 C). However, insolubility resulted in low material utilization. Therefore, a porous composite material containing 21 wt % of reduced graphene oxide (PFQ/rGO) was synthesized, which had better capacity utilization. Recently, our^29^, ^31^, ^35^ and other research groups^32^, ^36^, ^37^, ^38^ have shown that redox‐active organic materials can be successfully used as Mg cathode materials. The advantage of organic materials in Mg battery systems can be attributed to their relatively flexible structure and swelling of organic materials inside the electrolyte, which allow better accessibility to the Mg ions. Additionally organic materials have lower redox‐potentials that fit well inside the Mg electrolyte stability window.

Herein, we compared the redox reaction mechanism of PFQ/rGO in Li‐ and Mg‐metal organic batteries. The redox mechanism in the Li battery was probed by collecting operando ATR‐IR spectra during charge/discharge of the Li‐PFQ/rGO battery. Then, DFT calculations and IR spectra of the chemically synthesized monomer molecules were used to accurately assign bands from the operando measurements and assist their interpretation. PFQ/rGO was also used as an Mg battery cathode and its mechanism was analyzed with the help of ATR‐IR and DFT.

## Results and Discussion

### Li‐PFQ/rGO battery

**Figure 1:**
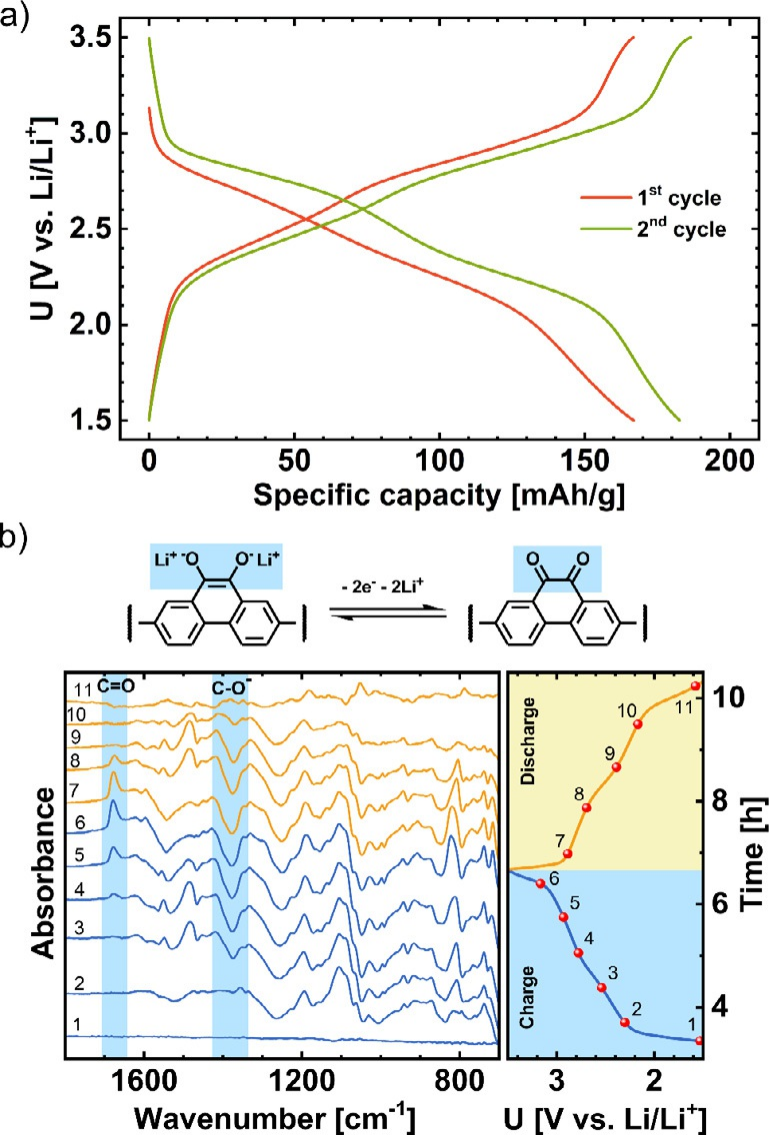
(a) Galvanostatic cycling of Li‐PFQ/rGO battery at a 0.2 C current density in 1 m LiTFSI in DOL+DME electrolyte. (b) Operando subtraction ATR‐IR spectra of the 2^nd^ charge and discharge.

Galvanostatic curves of the Li‐PFQ/rGO battery are displayed in Figure 1 a. PFQ/rGO had two charge plateaus at 2.47 and 2.93 V and two discharge plateaus at 2.78 and 2.26 V vs. Li/Li^+^, which were more clearly visualized by obtaining a dQ/dE plot (Figure S1). Two plateaus and a reversible capacity well above 130 mAh g^−1^ (130 mAh g^−1^ is the theoretical capacity for a one‐electron reaction) suggested that a two‐step redox reaction occurred. In each step, one electron should be exchanged. To confirm this hypothesis, we performed operando ATR‐IR measurements during cycling of the Li‐PFQ/rGO battery. For this measurement, we used a special spectro‐electrochemical pouch cell with a Si wafer window, which was in contact with Ge ATR crystal. For this operando ATR‐IR setup, the penetration depth was estimated to be 0.4–1.20 μm in the measurement region 1800–600 cm^−1^.^39^ The estimated thickness of our electrodes was approximately 150 μm, which means that only a small part of the electrode was probed. In the case of the insoluble PFQ polymer, in which the cathode active material does not dissolve, this is not a problem. A strong IR signal from the Si window and electrolyte was subtracted according to the literature.^39^, ^40^, ^41^ Subtraction visualizes only IR‐active changes inside the cathode composite during cycling (Figure 1 b). For the background, we used IR spectra of the discharged cathode. A negative band in the operando spectra means a decrease, and a positive band means an increase in the concentration of the corresponding species compared with the discharged state. During charging, the intensity of the carbonyl band C=O at 1678 cm^−1^ increased, whereas the corresponding band of the C−O^−^ group located at 1377 cm^−1^ decreased. During discharging, the process was reversed, indicating a reversible electrochemical redox reaction. These results indicated that the redox mechanism was reversible oxidation of the Li hydroquinone salt into quinone. These results were very analogous to operando ATR‐IR measurement of a comparable compound PAQS (C=O stretching at 1670 cm^−1^, and 1650 cm^−1^ and C−O^−^ stretching at 1370 cm^−1^).^40^ Besides these two bands, we observed many other positive/negative bands, which are analyzed in detail in the following section.

The assignment of the IR bands in the operando ATR‐IR spectra was achieved by comparison with the theoretical spectra calculated by DFT and the IR spectra of the chemically synthesized monomer molecules. First, the accuracy of the DFT calculations was verified by calculating the FTIR spectra of the chemically synthesized monomer molecules 2,7‐dibromo‐9,10‐phenanthrenequinone (FQ), dilithium salt of 2,7‐dibromo‐9,10‐dihydroxy‐phenanthrene (LiFQ), and the magnesium salt of 2,7‐dibromo‐9,10‐dihydroxyphenanthrene (MgFQ). These calculated spectra were compared with measured ones (Figure S2 a–c). The spectra matched surprisingly well, especially for the most intense bands such as the carbonyl C=O stretching, C=C and C−O^−^ stretching vibrations, ring vibrations and C−H out‐of‐plane deformations. However, some bands were shifted. The biggest mismatch was observed for the carbonyl band, for which the calculated frequency was 65 cm^−1^ higher than the measured value (Figure S2 a). Inaccurate intensities of the bands were also calculated by DFT, especially at lower frequencies; higher intensities were observed by operando ATR measurements at lower frequencies. Because of these two reasons, we decided to zoom in on the calculated DFT spectra 3–6× times in the region below 1300 cm^−1^ to obtain more comparable results with operando ATR measurements. Notably, the calculations revealed the expected complex nature of the bands that belong to the ring vibrations. Those bands were the combination of C−C, C=C, C−H, and C−O^−^ or C=O modes that were strongly coupled. The appearance of such coupled vibrations can be an additional source of the observed differences between the calculated and measured spectra.

**Figure 2:**
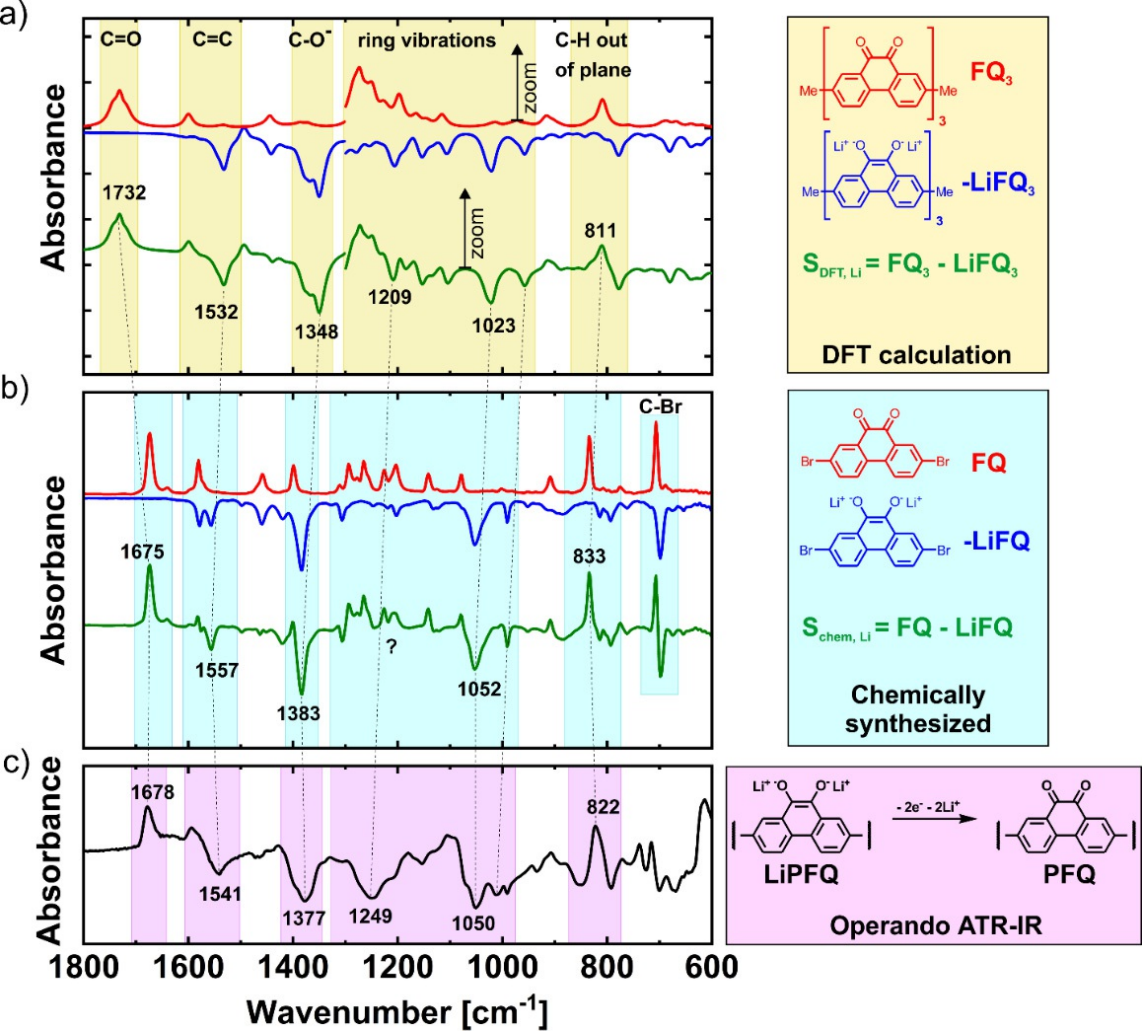
Comparison of (a) DFT calculation of model trimer, 3–6× zoom was used below 1300 cm^−1^, (b) measured IR spectra of chemically synthesized reference monomer and (c) operando ATR‐IR spectra of a Li‐PFQ/rGO battery at the end of charge at 3.5 V vs. Li/Li^+^.

In the next step, DFT calculations were applied for LiFQ_3_ and FQ_3_ oligomers (Figure 2 a), which consist of three monomer units. Using larger oligomers as models would give us more accurate predictions but would significantly extend the time required to perform the calculations. The subtracted DFT spectra were calculated using the equation S_DFT, Li_=FQ_3_−LiFQ_3_. For comparison, we also calculated the subtracted spectra for the chemically synthesized reference monomers S_chem, Li_=FQ−LiFQ (Figure 2 b), which were very similar to the operando ATR‐IR spectra (Figure 2 c). For instance, the carbonyl C=O stretching band^42^, ^43^ at 1678 cm^−1^ from operando ATR‐IR measurement was very close to the band observed for the chemically synthesized monomer at 1675 cm^−1^. The C−O^−^ stretching vibration at 1377 cm^−1^ was close to the predicted value of 1348 cm^−1^ and the measured value of 1383 cm^−1^ or the chemically synthesized monomers. Other bands are explained in the Supporting Information and in Table S1.

**Figure 3:**
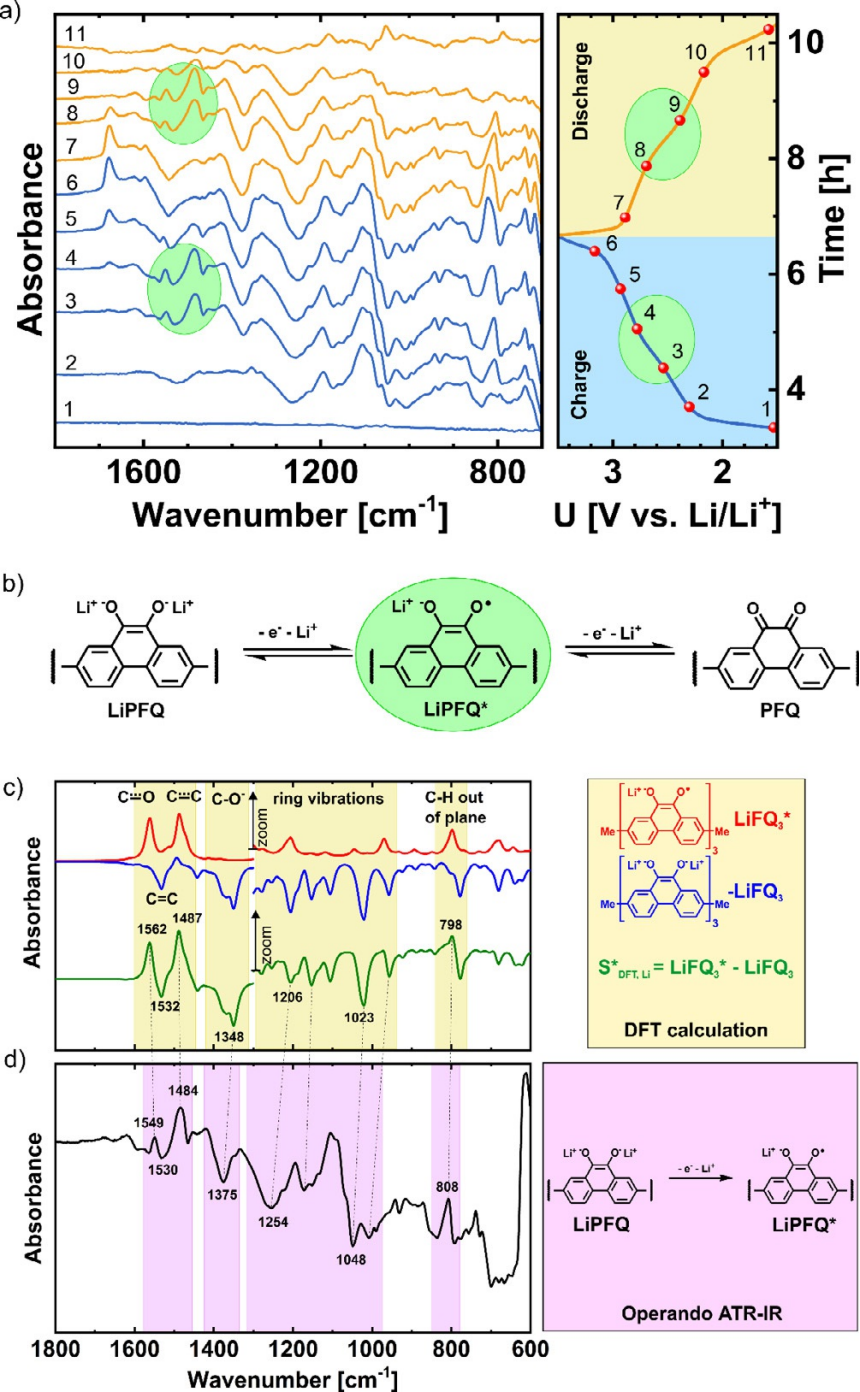
(a) Operando ATR‐IR spectra of the Li‐PFQ/rGO battery during charging/discharging. (b) Proposed redox reaction with intermediate radical anion LiPFQ*. (c) DFT spectra of the radical anion trimer LiFQ_3_*, discharged state LiFQ_3_, and their subtraction spectrum. 3–6× zoom was used below 1300 cm^−1^. (d) *Operando* ATR‐IR spectra in the middle of the charge at 2.67 V vs. Li/Li^+^.

From the charge/discharge galvanostatic curves, two plateaus were easily distinguished, which was attributed to a two‐electron reaction in two separate one‐electron steps. Using the operando ATR‐IR technique (Figure 3 a), we detected the intermediate radical anion LiPFQ* (Figure 3 b). Two bands at 1549 and 1484 cm^−1^ started to appear towards the middle of the charge/discharge cycle and were in good agreement with the bands at 1562 and 1487 cm^−1^ calculated by DFT (Figure 3 c and 3 d); they corresponded to the stretching vibration of the C${}^{\underline{.....}}$ O and C${}^{\underline{.....}}$ C bonds. The position of those bands was in good agreement with the data in the literature.^44^ Other bands monotonically increased during charging and corresponded to C−O^−^ (1375 cm^−1^), ring vibrations (1254 cm^−1^ and 1048 cm^−1^), and C−H out‐of‐plane vibrations (808 cm^−1^).

### Mg‐PFQ/rGO battery

**Figure 4:**
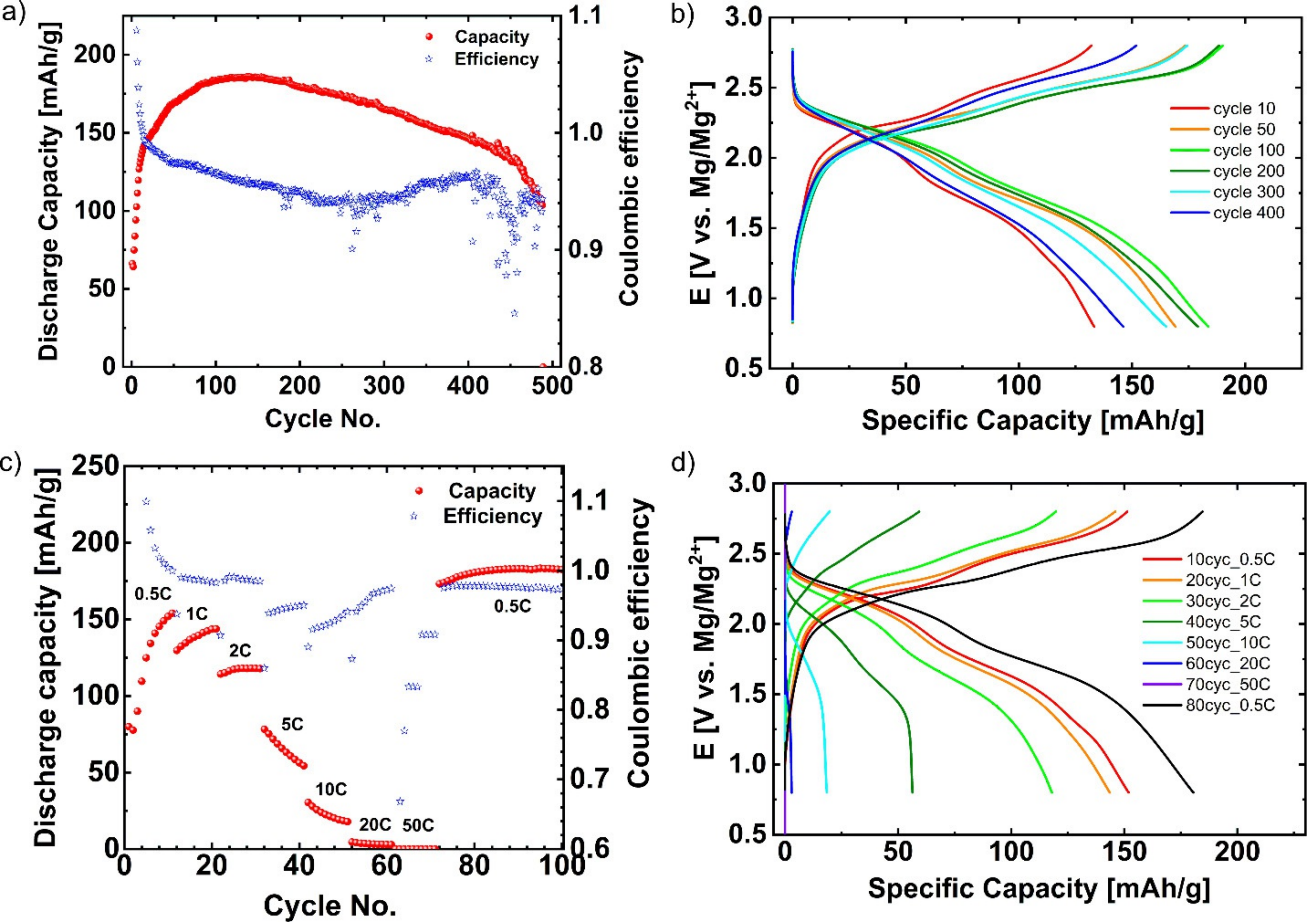
Cycling of the Mg‐PFQ/rGO battery under: (a) at 0.5 C for 490 cycles (0.8–2.8 V vs. Mg/Mg^2+^), (b) galvanostatic curves at different cycles numbers at 0.5 C, (c) rate capability test from 0.5 C to 50 C (0.8–2.8 V vs. Mg/Mg^2+^), (d) galvanostatic curves at different current densities from 0.5 C to 50 C.

The electrochemical performance of the PFQ/rGO composite material in an Mg battery was evaluated using 0.6 m Mg(TFSI)_2_‐2MgCl_2_ in dimethoxyethane (DME) electrolyte at 0.5 C current density. The initial capacity was only 65 mAh g^−1^, but it quickly increased to 130 mAh g^−1^ after only ten cycles (Figure 4 a). The subsequent increase to 186 mAh g^−1^ (72 % of the theoretical capacity *C*_theo_=260 mAh g^−1^) was very gradual. Between 140–400 cycles, the capacity slowly decreased to 146 mAh g^−1^ (8 % drop/100 cycles). To the best of our knowledge, this is one of the best results for an Mg battery in terms of discharge capacity and cycle stability. The Coulombic efficiency in the first 14 cycles was above 100 % owing to large polarization in the first cycles, which prematurely finishes the discharge. Consequently, some of the initial capacity (pristine material was in a charged state), was retained and the discharge capacity was larger than the charge capacity in the previous charge. The efficiency with cycling dropped to 94 % after 250 cycles and later increased back to 96 % until the 400^th^ cycle. After 400 cycles, sporadic Coulombic efficiency drops were observed and the capacity drop was more prominent. The most likely reason for this was degradation of the Mg anode. As shown in the galvanostatic curves, the polarization decreased until 100–200 cycles (Figure 4 b). At the 100^th^ cycle, two discharge plateaus at 2.19 and 1.71 V vs. Mg/Mg^2+^, and two charge plateaus at 2.25 and 2.55 V vs. Mg/Mg^2+^ were observed and more clearly visualized with a dQ/dE plot (Figure S3). Compared with the results of the Li system, the equilibrium potential was only approximately 420 mV lower in the Mg system and the expected value was approximately 700 mV (*E*_(Li/Li+)_−*E*_(Mg/Mg2+)_=3.040 V−2.372 *V=*668 mV). A higher potential in the Mg battery system could be explained by an energetically more favorable coordination of the Mg^2+^ ion with two C−O^−^ in the ortho position compared with two Li^+^ ions.^35^, ^45^ We also tested the C‐rate performance of the Mg‐PFQ/rGO battery from 0.5 C to 50 C rate (Figure 4 c). Owing to a slow activation, the capacity was stabilized after 32 cycles (at 2 C). However, we could still compare the capacities relative to 0.5 C at the end of the cycling (183 mAh g^−1^, 100 cycles). At a current density of 5 C, the capacities decreased to approximately one third and at >20 C no capacity was obtained. The Coulombic efficiency during the rate capability tests decreases with higher C‐rate from approximately 99 % to 83 %, which is rather unusual—in most cases the efficiency improves at high rates because there is less time for side reactions. In our case, there may be a problem with premature cut‐off at high rates, at which polarization becomes significant. More detailed information about the C‐rate experiments was obtained from galvanostatic curves (Figure 4 d). The polarization increased with higher C‐rate, which was expected. The capacity drop was mainly attributed to the charge cut‐off potential—the charging plateau at 5 C and higher rates was finished too early at 2.8 V vs. Mg/Mg^2+^. Therefore, the capacity at high rates could be improved by setting the cut‐off voltage to higher values, but this is limited by the electrolyte stability window, which is approximately 2.8 V vs. Mg/Mg^2+^ for high‐surface‐area electrodes. The low capacity in the early cycles can also be explained in the same manner: The material could not be fully charged in the first cycles until the polarization gradually drops to allow full charging of the material.

**Figure 5:**
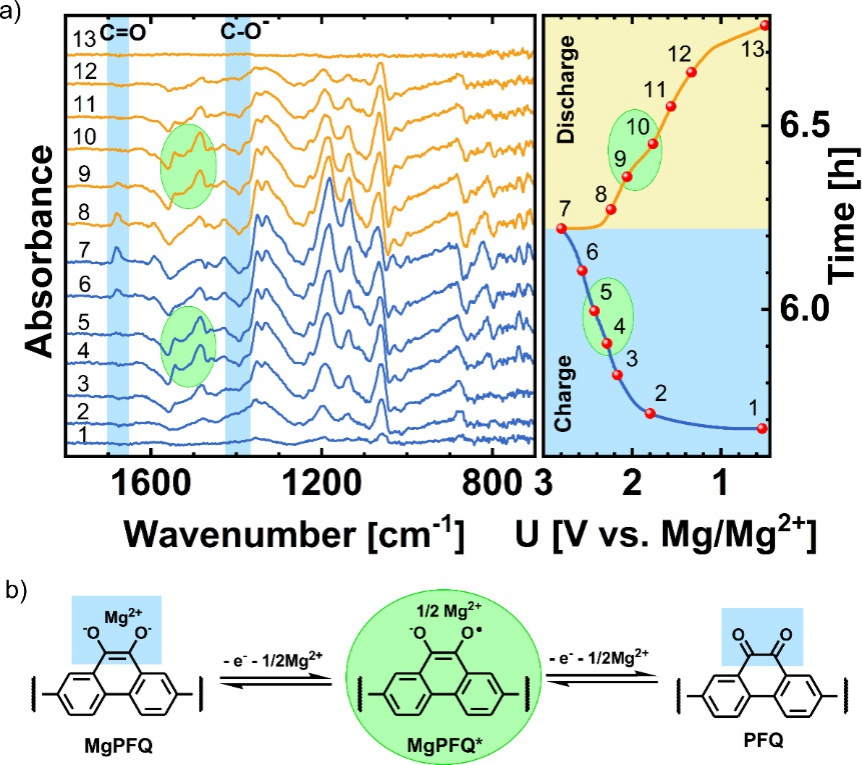
(a) Operando ATR‐IR spectra of PFQ/rGO during cycling of Mg‐PFQ/rGO battery at 0.5 C current density and (b) proposed redox mechanism of PFQ during charge/discharge.

The operando ATR‐IR spectra during cycling of the Mg‐PFQ/rGO battery were obtained in a similar manner as for the Li battery system. During charging, the carbonyl C=O band at 1680 cm^−1^ increased and the C−O^−^ band at 1392 cm^−1^ decreased, although both were much smaller compared with those in the Li system owing to a lower capacity utilization (Figure 5 a). However, we proposed a redox reaction mechanism in which the Mg hydroquinone salt MgPFQ was reversibly oxidized into quinone PFQ via a radical anion MgPFQ* (Figure 5 b).

**Figure 6:**
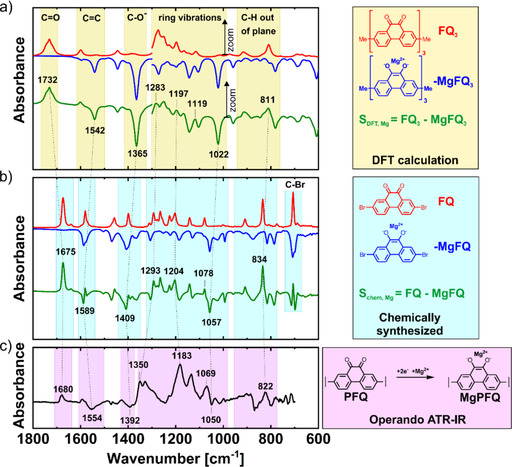
Comparison of (a) DFT calculation of model trimer, 3–6× zoom was used below 1300 cm^−1^, (b) measured IR spectra of chemically synthesized reference monomer and (c) operando ATR‐IR spectra of Mg‐PFQ/rGO battery at the end of charge at 2.8 V vs. Mg/Mg^2+^.

We used the same strategy as for the Li system to assign the bands (Figure 6 a and 6 b). The characteristic bands for C=O, C=C, C−O^−^, and C−H out‐of‐ring vibrations were at similar positions as in the Li system (Table S1). On the other hand, the ring vibrations from 1350 to 1000 cm^−1^ in the Mg system were quite different compared with the Li‐system. The same pattern was observed in this region in the spectra obtained by DFT calculations and for the chemically synthesized monomers: two bands (1350 cm^−1^ and 1330 cm^−1^), followed by three bands (1183 cm^−1^, 1135 cm^−1^ and 1069 cm^−1^), and a negative band at 1050 cm^−1^.

**Figure 7:**
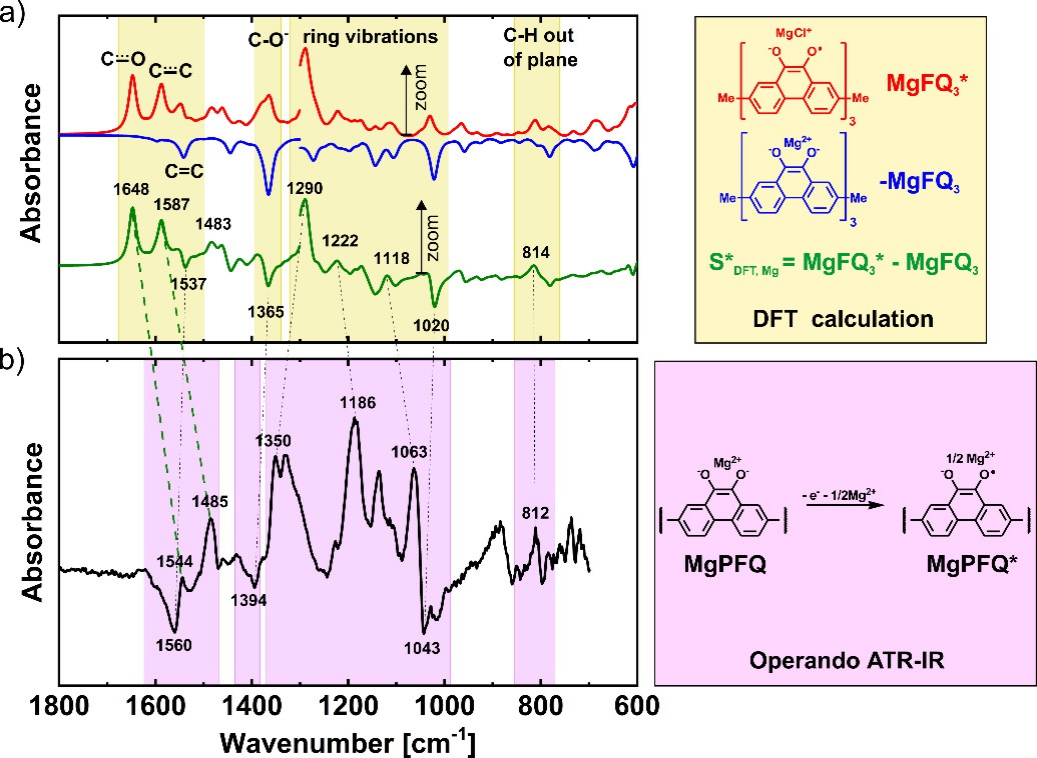
(a) DFT calculation of the model trimer: radical anion MgFQ_3_*, discharged state MgFQ_3_, and their subtraction spectrum. 3–6× zoom was used below 1300 cm^−1^. (b) Operando ATR‐IR spectra in the middle of the charge at 2.36 V vs. Mg/Mg^2+^.

Two plateaus were also observed in the Mg system during charging/discharging, suggesting that a two‐electron redox reaction has two separate processes with an intermediate relatively stable radical anion (Figure 5 b). Operando ATR‐IR spectroscopy was used to confirm this hypothesis (Figure 5 a). Again, two characteristic bands at 1544 and 1485 cm^−1^ were observed, corresponding to C${}^{\underline{.....}}$ O and C${}^{\underline{.....}}$ C vibrations of an anion radical. However, a large difference between the measured and calculated values (1648 cm^−1^ and 1587 cm^−1^) was observed (Figure 7). One of the reasons could be because we were not able to use 1/2 Mg^2+^ ion per monomer unit in the DFT calculations;. the MgCl^+^ ion was used instead. The use of MgCl^+^ was justified by the fact that in Mg battery systems with bis(trifluoromethane)sulfonimide magnesium salt or Mg(TFSI)_2_ and MgCl_2_‐based electrolytes, the cathode material is exchanging both Mg^2+^ and MgCl^+^.^39^, ^46^ All other bands were also present in the charging spectra (Figure 6 c, Table S1) and they were slightly shifted.

## Conclusions

PFQ/rGO active material is a next generation cathode material with extraordinary cycling stability in both Li‐ and Mg‐based organic batteries. In a Mg system, the material displays high capacity utilization of 186 mAh g^−1^ and a small capacity fade 0.08 % per cycle with an average discharge potential of 1.8 V vs. Mg/Mg^2+^. The rate capability of Mg‐PFQ/rGO was moderate, which was mainly attributed to relatively high polarization of the Mg metal anode. Starting with the Li electrolyte, the electrochemical mechanism of PFQ/rGO was investigated through operando ATR‐IR. Operando ATR‐IR spectra were complemented with the synthesis of model compounds and DFT calculations, which confirmed the quinone/hydroquinone salt electrochemical mechanism. The formation of a radical anion was identified as an intermediate step through the appearance of new bands at 1549 and 1484 cm^−1^_,_ which were the most intense at the middle of the half cycle and assigned with the help of DFT calculations. Interestingly, bands of the radical anion were also observed in the Mg system, which might indicate a complex electrochemical mechanism with the possibility of MgCl^+^ or other complex monovalent ions serving as counterions. Another option is that Mg^2+^ ions interact with two neighboring PFQ radical ions. Nevertheless, the PFQ/rGO cathode showed promising performance as a cathode in an Mg battery, which opens a path towards its application in other multivalent battery systems (e.g., Al, Zn, Ca). Its high cycling stability might lead to practical multivalent organic batteries.

## Experimental Section

### Poly(phenanthrene quinone)/graphene composite (PFQ/rGO)

Phenanthraquinone was brominated by using *N*‐bromosuccinimide (NBS) in H_2_SO_4_ to obtain a brominated derivative of monomer 2,7‐dibromo‐9,10‐dihydroxyphenanthrene (FQ). Then FQ was reduced to 2,7‐dibromo‐9,10‐dihydroxyphenanthrene with Sn/HCl, which was further acetylated to monomer 9,10‐diacetoxy‐2,7‐dibromophenanthrene. The latter was polymerized using bis(cyclooctadiene)nickel(0) or Ni(COD)_2_ in the presence of reduced graphene oxide and dispersed in *N*‐methyl pyrrolidone. The as‐obtained polymer composite was deprotected with LiAlH_4_ and then oxidized using 2,3‐dichloro‐5,6‐dicyanobenzoquinone (DDQ) to obtain the PFQ/rGO polymer composite. More information is available in the literature.^34^

### 2,7‐dibromo‐9,10‐phenanthrenequinone (FQ)

9,10‐Phenanthrenequinone (7.00 g, 33 mmol) was brominated using NBS (12.86 g, 72 mmol) in 190 mL of concentrated sulfuric acid (97 %) at room temperature for 8 h. The impure product (12.68 g, 103 % yield) was further purified by crystallization in boiling dimethylformamide (120 mL), filtrated, washed two times with cold dimethylformamide and dried overnight in an oven at 50 °C to obtain a yellow crystalline product (8.13 g, 66 % yield). More information is available in the literature.^47^

### dilithium salt of 2,7‐dibromo‐9,10‐dihydroxyphenanthrene (LIFQ)

2,7‐dibromo‐9,10‐phenanthrene‐quinone (FQ) (3.66 g, 10 mmol) was reduced using metallic tin (4 g, 34 mmol) in a mixture of 10 mL of concentrated HCl and 50 mL glacial acetic acid under reflux for 1 h to yield reduced 2,7‐dibromo‐9,10‐dihydroxyphenanthrene as a colorless crystalline solid (3.64 g, 99 % yield) according to the literature.^34^, ^48^ ^1^H NMR (300 Hz, [D_6_]DMSO): *δ*=9.40 (2 H, s), 8.67 (2 H, d, *J=*8.9 Hz), 8.30 (2 H, d, *J=*2.1 Hz), 7.66 ppm (2 H, dd, *J=*8.9, 2.1 Hz). ATR‐IR: $\tilde{\nu }$ =3371, 1630, 1598, 1435, 1302, 1200, 1123, 1031, 928, 880, 792, 691 cm^−1^. Reduced 2,7‐dibromo‐9,10‐dihydroxyphenanthrene (30 mg, 0.082 mmol) was dissolved in dry tetrahydrofuran (2 mL). Then, a solution of 2.5 m *n‐*butyl lithium in hexanes (Sigma Aldrich, 66 μL, 0.164 mmol, 2 equiv.) was slowly added to the above solution. The color first changed from yellowish to dark green, then to orange at the end of the addition. The reaction mixture was stirred for 1 day at 30 °C, then the volatiles were removed at 50 °C under reduced pressure to obtain an orange powder (32 mg, 103 % crude yield). All the reaction steps, isolation, and characterization were performed inside a glovebox under an inert Argon atmosphere as these salts can be very easily oxidized in an air atmosphere. ^1^H NMR (300 MHz, [D_6_]DMSO): *δ*=8.52 and 8.33 (4 H, br m), 7.07 ppm (2 H, br s), ATR‐IR: $\tilde{\nu }$ =2975, 2957, 2875, 1579, 1557, 1460, 1420, 1384, 1306, 1203, 1053, 991, 885, 814, 794, 699 cm^−1^.

### Magnesium salt of 2,7‐dibromo‐9,10‐dihydroxyphenanthrene (MgFQ)

MgFQ was prepared in a similar manner to the Li salt above (LiFQ), with the only difference the addition of 1 m *n*‐dibutyl‐Mg in heptanes (Sigma Aldrich, 82 μL, 0.082 mmol, 1 equiv.), was used instead of *n‐*butyl lithium (33 mg, 104 % crude yield). ^1^H NMR (300 Hz, [D_6_]DMSO): *δ*=8.57 (2 H, d, *J=*2.3 Hz), 8.39 (2 H, d, *J=*8.8 Hz), 7.15 ppm (2 H, dd, *J=*8.8, 2.3 Hz). ATR‐IR: $\tilde{\nu }$ =2954, 2869, 1587, 1469, 1407, 1365, 1307, 1186, 1130, 1057, 1025, 994, 876, 816, 786, 708 cm^−1^.

### ATR‐IR spectra

Infrared spectra of the reference monomers FQ, LiFQ, and MgFQ were measured inside a glovebox under an inert Argon atmosphere on an IR spectrophotometer Bruker Alpha using Germanium ATR crystal. The measuring range was 4000–600 cm^−1^. 32 consecutive scans were measured with a resolution 4 cm^−1^.

### Operando ATR‐IR spectroscopy

The ATR‐IR measurements were performed on a Bruker Vertex 80 equipped with a Specac Silver Gate Ge crystal ATR and a liquid‐nitrogen‐cooled mercury cadmium telluride (MCT) detector. The spectra were collected in absorbance mode with 64 scans at a resolution of 4 cm^−1^ in the range from 4000 cm^−1^ to 600 cm^−1^. The IR spectra were collected in operando mode with a series of repetitive scans every 2 min during galvanostatic cycling of the batteries with a current density of 0.2 C or C/5 rate for the Li battery system and 0.5 C for the Mg battery system. The battery was assembled in a spectro‐electrochemical cell with a Si wafer window.^39^, ^40^ The atmospheric compensation was performed on the IR spectra in OPUS version 7.8 software. The ATR difference spectra were obtained by subtracting the discharge spectrum from the obtained IR spectrum at a specific point of the discharge/charge.

### DFT calculations

DFT calculations were performed using the Gaussian 09 software package.^49^ The M06‐2X hybrid functional^50^ and 6‐31+G(d,p) basis sets were used to do geometry optimization and calculation of the vibrational frequencies and IR intensities. To model the polymer PFQ and its reduced forms in the Li and Mg battery system, a trimer consisting of three monomer units that end with a methyl group was used (FQ_3_, LiFQ_3_, LiFQ_3_*, MgFQ_3_, and MgFQ_3_*) in all cases (Figure S4). The models ensured suitable accuracy of the calculation while keeping the computational cost reasonable. Vibrational modes of methyl groups that are included in the model owing to the finite size of the trimer, are not included in the analysis and plotted spectra. To account for the effects of the surrounding environment, a dielectric constant of 7.4 was used, which is a common value for ether‐based electrolytes. Discrepancies between the theoretical and experimental spectra arise owing to approximations of the theoretical approach, such as the treatment of the electronic Hamiltonian, finite basis set, and the harmonic approximation. This leads to overestimation of the calculated frequencies by approximately 5 %. Therefore, we applied a scaling factor of 0.95, which allows easier visual comparison between the theoretical and experimental values.

### NMR spectra

NMR spectra were collected using a 300 MHz Varian Unity Inova. Deuterated solvents CDCl_3_ or [D_6_]DMSO were used and tetramethyl silane was used as a standard.

### Assembly of Li batteries

Electrodes were prepared by mixing 60 mg of composite PFQ/rGO, 30 mg of carbon black (Printex XE2), and 10 mg of polytetrafluoroethylene (PTFE) (60 wt % water dispersion, Aldrich) and 0.5 mL of isopropyl alcohol (IPA). All these ingredients were ball milled in 12 mL stainless steel grinding jars (10 mm Ø balls) with a planetary ball mill (Retsch PM100) at 300 rpm for 30 min in an air atmosphere. The obtained slurry was kneaded with a mortar and pestle to obtain a compact black gum. The gum was rolled between two pieces of a nonadhesive paper with a roller to obtain an electrode film of approximately 5×5 cm size. An aluminum mesh (100 mesh size) was deposited on top of this film and the film was rolled again to glue the electrode composite and the mesh together, and then dried in an air atmosphere. Afterward, electrode discs with a diameter of 1.2 cm were cut and pressed with a load of 1 ton and further dried at 80 °C in vacuum for 1 day. The average loading on the electrode was 2.5±0.4 mg of active material per cm^2^. Battery cells were assembled in an argon‐filled glovebox (water and oxygen levels <1 ppm). Swagelok‐type battery cells were assembled using the above‐mentioned electrodes, a 13 mm glass fiber separator (Whatman GF/A), and freshly rolled lithium (12 mm diameter, Aldrich). 1 m Bis(trifluoromethane)sulfonimide lithium salt (Aldrich) in a mixture of dry 1,3‐dioxolane/dimethoxyethane was used an electrolyte (1 m LiTFSI/DOL+DME).

### Assembly of Mg batteries

An electrode composite gum for Mg tests was prepared in the same way as that for Li. Then, the gum was rolled in between two sheets of nonadhesive paper and afterwards electrode discs with 1.2 cm diameter were cut out to give self‐standing electrodes. The obtained electrodes were subsequently dried at 80 °C in vacuum for 1 day and transferred into an argon‐filled glove box. An average loading on electrode was 2.5±0.4 mg of active material per cm^2^. Mg cells were assembled in Swagelok type battery cells using graphite disc as cathode current collector, 13 mm glass fiber separator (Whatman GF/A) and freshly brushed Mg foil (Gallium Sources, 99.95 %, 0.05 mm). 0.6 m Mg(TFSI)_2_‐2MgCl_2_ in DME was used as electrolyte.

### Electrochemical measurements

A potentiostat/galvanostat VMP3 (Bio‐Logic, France) was used at room temperature (25 °C) to perform the electrochemical measurements. Batteries were cycled between 1.5–3.5 V vs. Li/Li^+^ at a current density of approximately 0.2 C or C/5. Mg batteries were cycled from 0.8–2.8 V vs. Mg/Mg^2+^ at different C‐rates from 0.5 to 50 C.

## Conflict of interest

*The authors declare no conflict of interest*.

## Supporting information

As a service to our authors and readers, this journal provides supporting information supplied by the authors. Such materials are peer reviewed and may be re‐organized for online delivery, but are not copy‐edited or typeset. Technical support issues arising from supporting information (other than missing files) should be addressed to the authors.

Supplementary
