# Supplementary material for: Redox Mechanisms in Li and Mg Batteries Containing Poly(phenanthrene quinone)/Graphene Cathodes using Operando ATR‐IR Spectroscopy
Source: ChemSusChem. 2020 Mar 19;13(9):2328–36. doi: 10.1002/cssc.202000054 (PMC7317575; doi:10.1002/cssc.202000054)
Supplement: Supplementary file 1 — Supplementary [file CSSC-13-2328-s001.pdf]

ChemSusChem

Supporting Information

Redox Mechanisms in Li and Mg Batteries Containing Poly(phenanthrene quinone)/Graphene Cathodes using Operando ATR-IR Spectroscopy

Alen Vizintin+,[a] Jan Bitenc+,[a] Anja Kopač Lautar+,[a] Jože Grdadolnik,[a] Anna Randon Vitanova,[b] and Klemen Pirnat*[a]

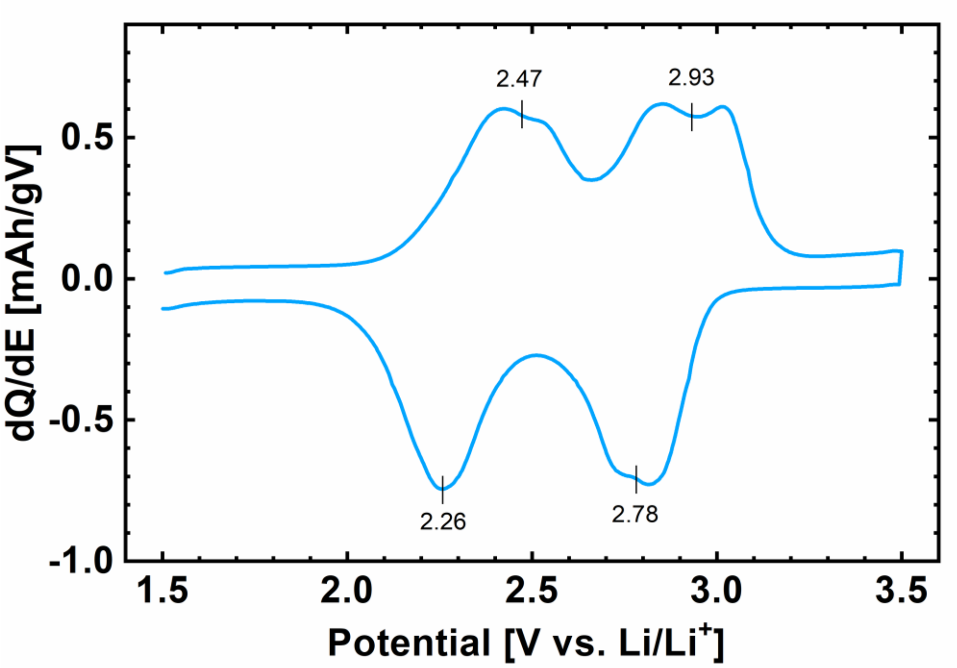

Figure S1. dQ/dE curve for Li-rGO/PFQ battery in 10th cycle at C/5.

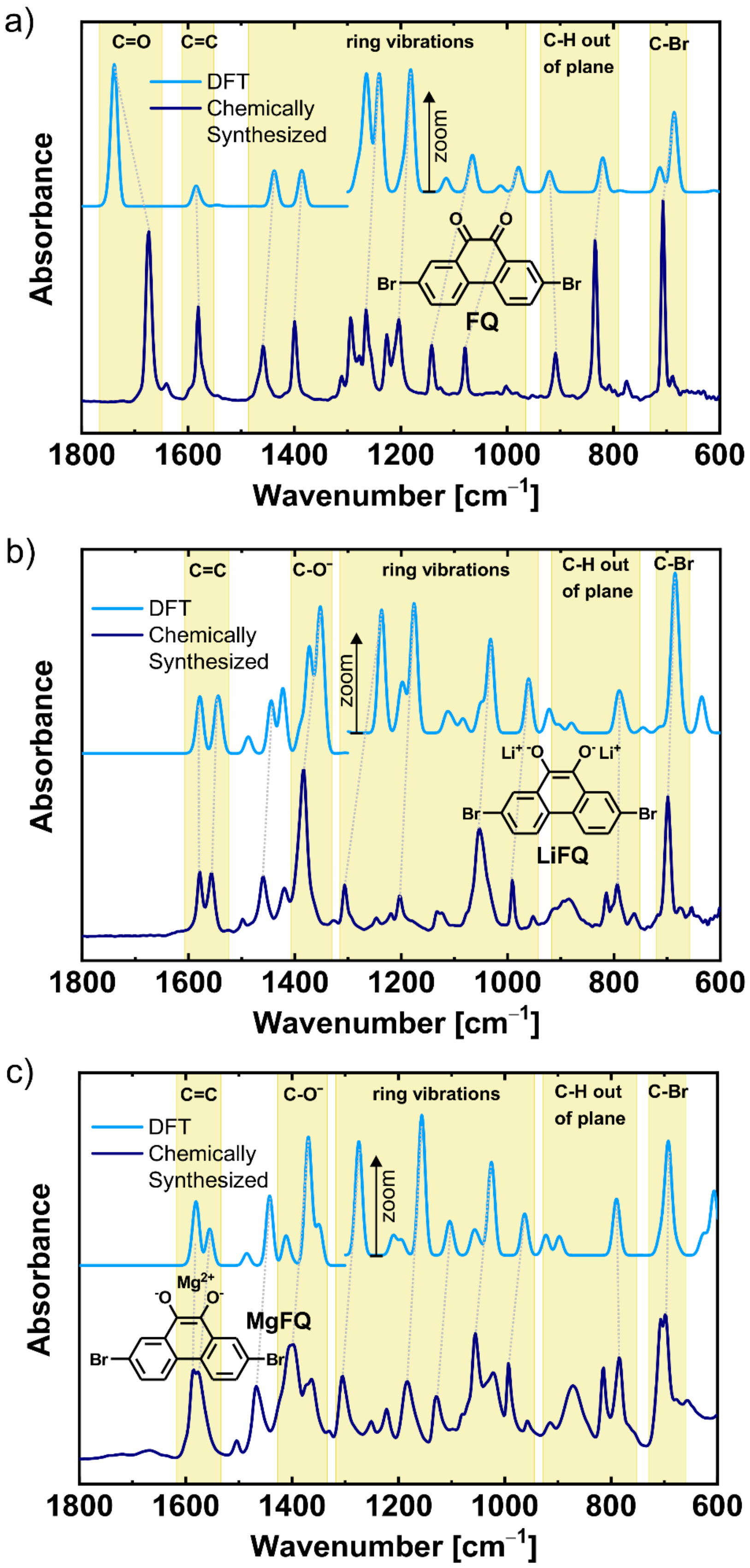

Figure S2. Theoretical and measured IR spectra for chemically synthesized reference monomer compounds FQ and LiFQ and MgFQ. 3-6× Zoom was used below 1300 cm^{-1}.

Other IR bands from Li-PFQ/rGO battery system

Band at 1541 cm^{-1} is attributed to C=C stretching. Additional bands are due to ring vibrations (a combination of C–C, C=C, C–O^{-}, and C=O stretching and C–H deformation). The difference between the calculated and measured spectra is rather big that brings the uncertainty of proper assignment. Thus, the origin of a strong negative band at 1249 cm^{-1} for *operando* ATR-IR measurement is unknown and is not present in $S_{chem,Li}$. However taking into account the $S_{DFT,Li}$ spectrum, we can reasonably assume this band is due to presence of the a reduced form of $LiFQ_3$ located at 1209 cm^{-1}. The only band from the region of ring vibrations with a good matching is the strongest one at 1050 cm^{-1}, which corresponds to 1023 cm^{-1} from DFT and 1052 cm^{-1} from chemically synthesized monomer. In the region of C–H out-of-plane deformations, there is one strong negative band at 822 cm^{-1}. C–Br band at around 700 cm^{-1} is present only in the reference monomer and is not relevant for DFT calculations and *operando* ATR-IR measurements.

Table S1. Characteristic IR bands obtained by 1) DFT calculations, 2) by measuring chemically synthesized reference monomers and 3) by *operando* ATR-IR measurements in Li- and Mg-PFQ/rGO battery.

System	Method	C=O [cm^{-1}]	C=C [cm^{-1}]	C⩦O [cm^{-1}]	C⩦C [cm^{-1}]	C–O^{-} [cm^{-1}]	Ring vibrations [cm^{-1}]				C–H out-of-plane [cm^{-1}]
**Li charge**	DFT calculation	1732	1532			1348		1209		1023	811
	Chemically synthesized	1675	1557			1383		?		1052	833
	operando	1678	1541			1377		1249		1050	822
**Li radical**	DFT calculation		1532	1562	1487	1348		1206		1023	798
	operando		1530	1549	1484	1375		1254		1048	808
**Mg charge**	DFT calculation	1732	1542			1365	1283	1197	1119	1022	811
	Chemically synthesized	1675	1589			1409	1293	1204	1078	1057	834
	operando	1680	1554			1392	1350	1183	1069	1050	822
**Mg radical**	DFT calculation		1537	1648	1587	1365	1290	1222	1118	1020	814
	operando		1560	1544	1485	1394	1350	1186	1063	1043	812
	Literature	1680–1650[a]	~1600, ~1500[b]	1538–1535[c]	1488[c]	>1260[d]					900–650[e]

Table comments:

[a] Typical values for anthraquinones[1,2]

[b] Typical values for phenanthrene[1,2]

[c] Typical for protonated Semiquinone Radical Forms of Napthoquinones[3]

[d] 1260 cm^{-1} is for phenol. C–O^{-} in our case has higher frequency due to decrease in the mass of the vibrating C–OH group following its ionisation and partly due to the higher double bond character of the C–O^{-} bond, as compared with that of the original C–OH grouping.[4]

[e] Typical values for aromatic compounds.[1,2]

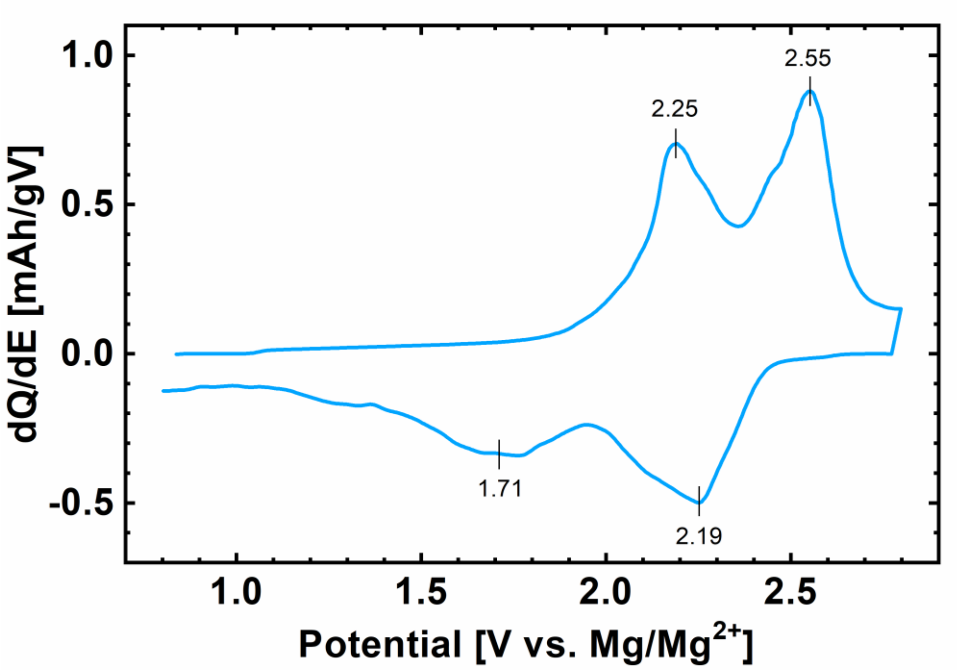

Figure S3: dQ/dE curve for Mg-rGO/PFQ battery in 100th cycle at 0.5C.

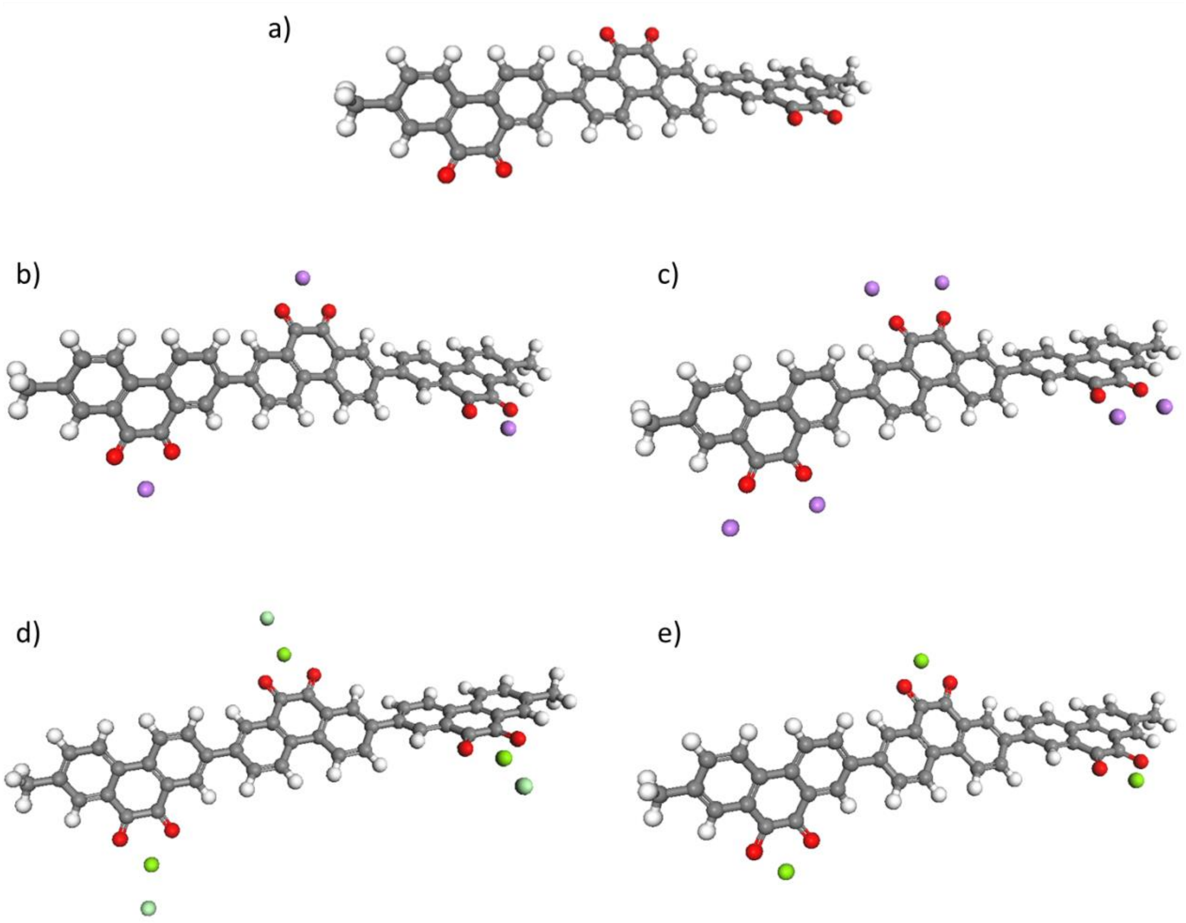

Figure S4. Trimer model for a) quinone FQ_3, b) Li radical $LiFQ_3^*$, c) Li salt $LiFQ_3$ d) MgCl used to model Mg radical $MgFQ_3$*, and e) Mg salt $MgFQ_3$. Atoms are marked with colors: O-red, H-white, C-grey, Li-purple, Mg-green, and Cl-pale green.

References

[1] J. B. Lambert, *Introduction to Organic Spectroscopy*, Macmillan, **1987**.

[2] G. Socrates, *Infrared and Raman Characteristic Group Frequencies*, John Wiley & Sons, LTD, West Sussex PO19 1UD, England, **2001**.

[3] J.-R. Burie, A. Boussac, C. Boullais, G. Berger, T. Mattioli, C. Mioskowski, E. Nabedryk, J. Breton, *J. Phys. Chem.* **1995**, *99*, 4059–4070.

[4] S. Pinchas, *Spectrochim. Acta Part A Mol. Spectrosc.* **1972**, *28*, 801–802.
